# Estimating the success of enzyme bioprospecting through metagenomics: current status and future trends

**DOI:** 10.1111/1751-7915.12309

**Published:** 2015-08-14

**Authors:** Manuel Ferrer, Mónica Martínez‐Martínez, Rafael Bargiela, Wolfgang R. Streit, Olga V. Golyshina, Peter N. Golyshin

**Affiliations:** ^1^ Institute of CatalysisConsejo Superior de Investigaciones Científicas (CSIC)Marie Curie 228049MadridSpain; ^2^ Biozentrum Klein FlottbekUniversität HamburgOhnhorststraße 18D‐22609HamburgGermany; ^3^ School of Biological SciencesBangor UniversityLL57 2UWGwyneddUK

## Abstract

Recent reports have suggested that the establishment of industrially relevant enzyme collections from environmental genomes has become a routine procedure. Across the studies assessed, a mean number of approximately 44 active clones were obtained in an average size of approximately 53 000 clones tested using naïve screening protocols. This number could be significantly increased in shorter times when novel metagenome enzyme sequences obtained by direct sequencing are selected and subjected to high‐throughput expression for subsequent production and characterization. The pre‐screening of clone libraries by naïve screens followed by the pyrosequencing of the inserts allowed for a 106‐fold increase in the success rate of identifying genes encoding enzymes of interest. However, a much longer time, usually on the order of years, is needed from the time of enzyme identification to the establishment of an industrial process. If the hit frequency for the identification of enzymes performing at high turnover rates under real application conditions could be increased while still covering a high natural diversity, the very expensive and time‐consuming enzyme optimization phase would likely be significantly shortened. At this point, it is important to review the current knowledge about the success of fine‐tuned naïve‐ and sequence‐based screening protocols for enzyme selection and to describe the environments worldwide that have already been subjected to enzyme screen programmes through metagenomic tools. Here, we provide such estimations and suggest the current challenges and future actions needed before environmental enzymes can be successfully introduced into the market.

## Introduction

Currently there is a great demand for suitable enzymatic biocatalysts that have high process performances and are ‘greener’ alternatives to chemical synthesis (Adrio and Demain, 2003; Fernández‐Arrojo *et al*., 2010; Bornscheuer *et al*., 2012; Turner and Truppo, 2013; Vergne‐Vaxelaire *et al*., 2013). It was expected that up to 40% of bulk chemical synthesis processes that now require environmentally damaging bulk organic solvents and elevated energy inputs could use enzymatic catalysis by 2030 (Adrio and Demain, 2003; Sawaya and Arundel, 2010; Zúniga *et al*., 2014). However, we have already surpassed the maximum rate of oil extraction (‘peak oil’), implying not only that we should look for sustainable sources of non‐fossil fuel but that we should also seek alternative ‘greener’ structural units within a molecule (synthons) for biopolymers and biomaterials (Timmis *et al*., 2014). Currently, the turnover of about USD 5 billion is produced by the application of enzymes in different markets (Sawaya and Arundel, 2010; Zúniga *et al*., 2014; and the World Enzymes to 2017 Report in http://www.rnrmarketresearch.com/world‐enzymes‐to‐2017‐market‐report.html), and the world enzyme demand is forecasted to rise from USD 6.4 to 6.9 billion p.a. in 2017. Accordingly, the demand for biocatalysts in the form of free or immobilized enzymes, whole cell catalysts or cell‐free systems, with a high applicability potential in industry is increasing (Schrewe *et al*., 2013; You and Zhang, 2013; Jeon *et al*., 2015; Schmidt *et al*., 2015).

The existing and recognized potential of environmental microbiology to substantially improve the commercial potential of biotechnology has recently been greatly strengthened by the advent of the molecular enzyme technology and metagenomics (Drepper *et al*., 2014). Although there is a breakthrough in protein design, and novel catalytic activities are now in reach that match those of natural enzymes (Woodley, 2013; Höhne and Bornscheuer, 2014), this technology provides the capacity to discover entirely new enzymes in microorganisms and their communities without the technically challenging need to culture them as individual species (Lee *et al*., 2010; Mora *et al*., 2011; Kyrpides *et al*., 2014; Yarza *et al*., 2014). In fact, Yarza and collegues (2014) provided an estimation of the uncultured microbial diversity. To date, only ∼ 11 000 bacterial and archaeal species have been described; however, at the current rate of ∼ 600 new descriptions per year, it has been predicted that it would take > 1000 years to classify all remaining microbial species. It thus remains unknown how long it would take to investigate the genomic information and enzymatic arsenals of these microbial species.

**Figure 1:**
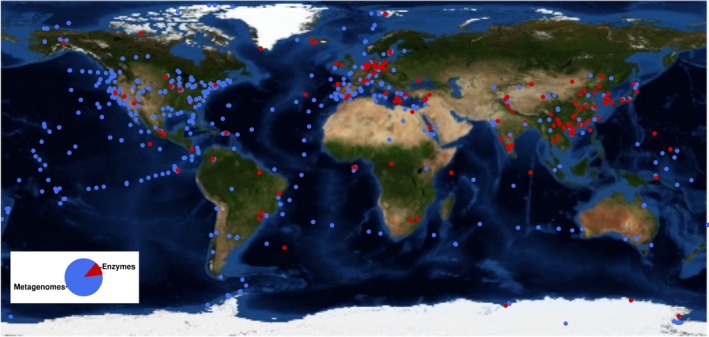
A survey of the metagenomic studies performed worldwide. The map has been created through the R language (2008) and the openstreetmap package (Eugster and Schlesinger, 2012) using the world map type ‘mapquest‐aerial’ and drawing the samples as points using the basic R tools. The figure is based on studies that were published over the last two decades and for which GPS coordinates were given. The databases used were SCOPUS, PubMed, WOK and the IMG/M webpage of the US Department of Energy Joint Genome Institute (http://www.jgi.doe.gov/). As shown, of the 2192 sites for which metagenomic studies (named ‘metagenomes’) have been reported (accounting only those for which GPS coordinates are available), only 256 (11.6%) were related to sites where enzymes or the clones containing them (red spots in the figure) have been isolated and partially characterized. As shown, only a tiny fraction of the sites have been subjected to studies on enzyme discovery from environmental resources. For details on sampling sites with indication of GPS coordinates, type of study (direct DNA sequencing or enzyme discovery) and habitat type, see Table S1.

**Figure 2:**
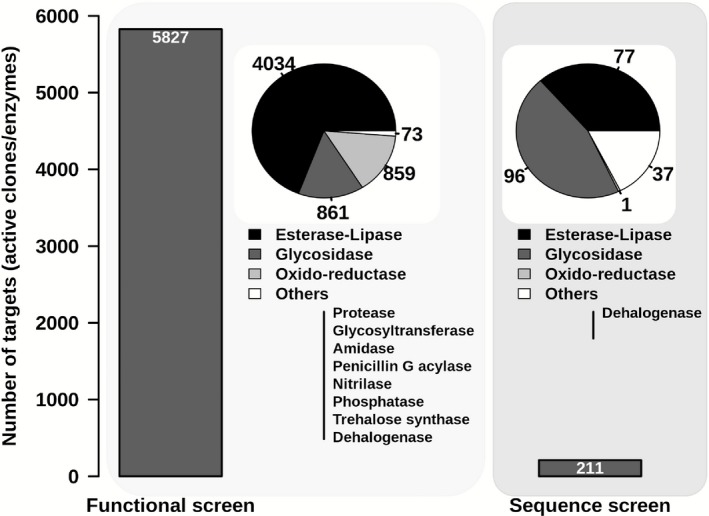
A survey of the total number of targets (clones and/or single enzymes and/or sequences encoding enzymes) identified by metagenomic studies. The distribution of selected targets as per enzyme activity type is shown per each of the two screening methods: naïve and *in silico* (sequence‐based) screens. The figure is based on studies that were published over the last two decades using naïve (left) and sequence‐based (right) screen protocols (see Table S1). The databases used to provide such estimations were SCOPUS, PubMed, WOK and the IMG/M of the US Department of Energy Joint Genome Institute (http://www.jgi.doe.gov/) and UniProtKB/Swiss‐Prot.

The metagenomic mining of enzymatic activities for biotechnological applications from microbial biodiversity (Niehaus *et al*., 2011), with an emphasis on microbes from extreme habitats, has recently been brought to a new technological level (Feller, 2013; Vester *et al*., 2014; Alcaide *et al*., 2015). However, despite the considerable progress made through the application of high‐throughput metagenomic sequencing and screening, the effective identification of existing enzymatic activities has only been completed in a rather limited number of environmental sites (Fig. 1 and Table S1). As an example, microbial communities from approximately 2192 different sites distributed across the planet have been examined for their metagenomic content. They include habitats such as terrestrial (topsoil, forest soil, plant rhizosphere soil, desert soil, Antarctic soil, compost, etc.), marine (tidal flat and coastal sediments, superficial and deep seawater, hydrothermal vents, etc.) and freshwater (pond water, etc.) habitats; other types of habitats included non‐marine saline and alkaline lakes, acid mine drainage systems, wastewater treatment sludges, compost (consortia bred on plant biomass) and eukaryotic‐associated microbiomes (marine sponge, termite and earthworms gut, shrimp gill, rumen, human microbiota, etc.) (for details, see Table S1). This suggests that we have apparently undersampled all representative types of habitats. Within the investigated sites, clones containing new enzyme activities or purified enzymes (a total of approximately 6100 described to date) were isolated and (mostly partially) characterized (Fig. 2) only in approximately 256 (or 11.6% of the total). Thus, although the global natural microbial diversity is known to be the major resource of new enzymes (Kyrpides *et al*., 2014; Yarza *et al*., 2014), this resource remains undersampled both at the level of habitats being explored and the number of new enzymes isolated from them.

## Bottlenecks in the metagenomic enzyme discovery process

The majority of metagenomics studies in the literature have identified enzyme variants that catalyse previously resolved reactions (Singh, 2010). There are very few cases in which a new enzyme has been translated into a process (Fernández‐Arrojo *et al*., 2010) or has shown reactivity (Alcaide *et al*., 2013) or physicochemical (Alcaide *et al*., 2015) properties that are significantly different from those previously reported. As example, an unusual ability to hydrolyse C‐O bonds in a broad spectrum of esters as well C‐C bonds in the aromatic ring fission products has been demonstrated for α/β hydrolases; they were isolated from crude‐oil enrichment cultures established with seawater and from a polycyclic aromatic hydrocarbon degrading bacterium (Alcaide *et al*., 2013). Also, moderately low temperature environments were shown to contain microbes with enzymes that are mostly active at temperatures as high as 70°C (Alcaide *et al*., 2015). However, there are some challenges in streamlining the transition from the discovery stage of an enzyme through its metagenomic analysis, and ultimately towards its end‐user applications (Jemli *et al*., 2014). The major technological bottlenecks include (i) a low proportion of coding metagenomic DNA accessible for expression (Guazzaroni *et al*., 2014), (ii) a low proportion of enzymes selected from screens perform well in industrial settings (Martínez‐Martínez *et al*., 2013), (iii) a lack of relevant substrates for screening (Fernández‐Arrojo *et al*., 2010), (iv) insufficient screening methods for rare enzymatic activities (Singh, 2010), (v) a poor performance of enzymes under non‐natural conditions (Fernández‐Arrojo *et al*., 2010), (vi) the existence of enzymes that are inactive after expression in the widely used host *Escherichia coli* (Loeschcke *et al*., 2013), (vii) the lack of reliable bioinformatics pipelines for analysis of next‐generation sequencing data generated from positive hits or direct sequencing (Nyyssönen *et al*., 2013), and (viii) the lack of reliable functional prediction of hypothetical proteins (Mende *et al*., 2012; Anton *et al*., 2013; Bastard *et al*., 2014; Chistoserdova, 2014). In addition, the minimization of amplification of annotation mistakes (sequence/activity incoherence) in databases (Fernández‐Arrojo *et al*., 2010) is among the more challenging issues to be solved. For example, using metagenomics approaches, Jiménez and colleagues (2012) reported a novel cold‐tolerant esterase; however, this protein was annotated in the database as a MarR family transcriptional regulator. This indicates that database entries are not fully reliable.

A number of corresponding solutions have been attempted or suggested. These include (i) the selective focusing on activity‐based enzyme mining, and the establishment of larger and diverse clone libraries (Alcaide *et al*., 2015), as well as the selective trapping of the activity‐encoding genes in two‐step selection processes (Yoon *et al*., 2007); (ii) the enrichment of environmental samples under conditions mimicking the application settings (Jiménez *et al*., 2014) and the consequent selection of microbes containing enzymes with high turnover rates under process conditions and industrial substrates; alternatively, harvesting of genes (through metatranscriptome analysis using cDNA sequencing approach) and proteins (through proteomic analysis) being most expressed under these conditions may also help in identifying not only highly active and novel enzymes but also those that can be expressed at high level, which is desired for their industrial productions (Akeroyd *et al*., 2013; Chang *et al*., 2013); (iii) prioritizing the screening and characterization of metagenomic sequences from uncultured microbes (Mackenzie *et al*., 2015) and single enzymes (Alcaide *et al*., 2013) with multiple activities, broad substrate spectra and stability across a broad range of physical and chemical conditions; (iv) the a‐la‐carte *de novo* synthesis of small molecules, chemical scaffolds and/or substrates (or dummies with functionalities similar to the target substrate of industrial interest) (Lim *et al*., 2013; Najah *et al*., 2013); in relation to this, the development of multi‐substrate approaches for high‐throughput functional screenings and/or design of new proxy chromogenic‐compounds that can mimic the real complex target substrates (Kračun *et al*., 2015) should be of high interest; (iv) the development of tailor‐made vectors and hosts for screening and expression (Loeschcke *et al*., 2013; Terrón‐González *et al*., 2013; Furubayashi *et al*., 2014; Liebl *et al*., 2014); (v) the *in silico* design and directed evolution of newly identified enzymes towards the most favourable biotechnological features (Brugger *et al*., 2014); (vi) the development of a computational workflow for gene discovery in full‐length inserts in positive clones and a protein product annotation system integrating state‐of‐the‐art and custom bioinformatics modules, with room for further refinements and improvements (Tasse *et al*., 2010; Schallmey *et al*., 2014) to generate hypothesis about enzyme functions in a similar fashion like in the Pfam database (Finn *et al*., 2014); and (vii) the development of an ‘unknown BLAST’ tool that implements the mapping of orthologous unknown enzymes (Ye and Doak, 2009; Anton *et al*., 2013).

## Quantifying the success of the screening protocols for enzyme discovery

Regardless of the advances in the above directions, enzymes can currently be efficiently identified and screened from metagenomic libraries or through homology searches in databases. In addition, the genomes of cultivable microbes or metagenomes are generally inspected for such enzymes that can be cloned and biochemically and structurally characterized (Lee *et al*., 2010; Hess *et al*., 2011; Kube *et al*., 2013).

The available literature on the application of high‐throughput screening methods in environmental clone libraries revealed that the production of readily screenable clone libraries poses a minimal challenge when searching for enzyme activities with high biotechnological potential and using simple substrates. In fact, a set of a few hundred enzymes can relatively easily be established within few months using a simple/single substrate. However, the incidence rate, or the measure of the frequency by which a positive clone with a desired activity occurs in the total screened clones (not the total number of clones in a library), depends on the enzyme activity under screening and the substrates used in the search, among other potential factors. Of note, the abundance level of the corresponding genes encoding the enzyme activities of interest in microbial genomes (see comments below) and the activity level of the enzymes are important factors affecting the efficiency of the screening programmes. Having said that, other key potential driving factors, such as the metagenome source, the DNA extraction method, the cloning vector, the expression system or host cells, the screening technique and screening conditions, to cite some, are additional factors influencing the success of the enzyme identification process. As example, enhanced expression systems based on viral components that prevent transcription termination at metagenomic terminators resulted in a sixfold increase in the frequency of carbenicillin resistant clones (Terrón‐González *et al*., 2013). Also, under the same screening conditions, the frequency of clones with carboxyl‐esterase activity varies from 1 each 667 to 1 each 15 000 clones when different deep‐sea habitats were examined (Alcaide *et al*., 2015).

**Figure 3:**
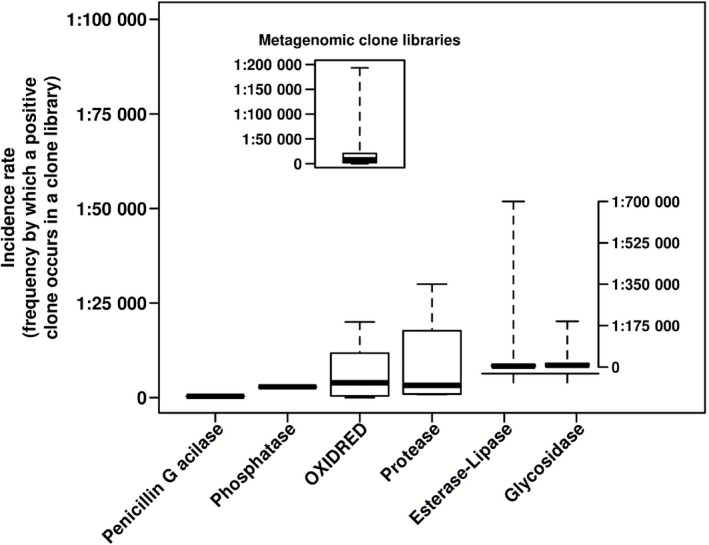
Box plots of the incidence rate of the positive clones (referred to the total number of clones screened) according to the enzyme activity. The results are based on the values for the metagenomic studies (see Fig. 1 legend) related to the top six activities commonly identified by naïve screens independent of the substrate used. The inset represents the mean incidence rate for all enzymes. Note: because the incidence rate depends on the type of clone library, only data regarding studies in which metagenomic fosmid clone libraries were screened were considered. OXIDRED, oxidoreductase. Data have been adapted from bibliographic records summarized in Table S1.

Common targets in metagenomic investigations are enzymes that are predominantly used in biocatalysis and industrial sectors (i.e. food, laundry, biofuels), such as acylases, phosphatases, proteases, oxidoreductases, glycosyl hydrolases and lipases/esterases (Fernández‐Arrojo *et al*., 2010). Other enzymes of industrial interest, such as nitrilases and transaminases, albeit being of industrial relevance (Bayer *et al*., 2011; Gong *et al*., 2013; Vergne‐Vaxelaire *et al*., 2013), have been scarcely examined by metagenomic approaches. For this reason, considering the most popular activity screens described in the specialized literature for those six industrially relevant types of enzymes, the following order could be established in relation to the mean incidence rate of positive clones when performing a naïve screen in the environmental clone libraries: acylases (1 active clone per 333 total clones; or 1:333), phosphatases (1:2843), oxidoreductases (1:6670), proteases (1:9388), esterase/lipases (1:17 320) and glycosidases (1:31 190) (Fig. 3). Note that these values are according to references provided in Table S1 for the 256 sites from which environmental enzymes have been isolated. In summary, the incidence rate for all of these activities has been shown to range from 1:11 to 1:193 200 (Fig. 3, inset), depending on the activity, substrate and habitat from which the library was constructed. Clearly, some activities are much more abundant than others (see comments below), and this should be considered when designing appropriate screening programmes.

**Figure 4:**
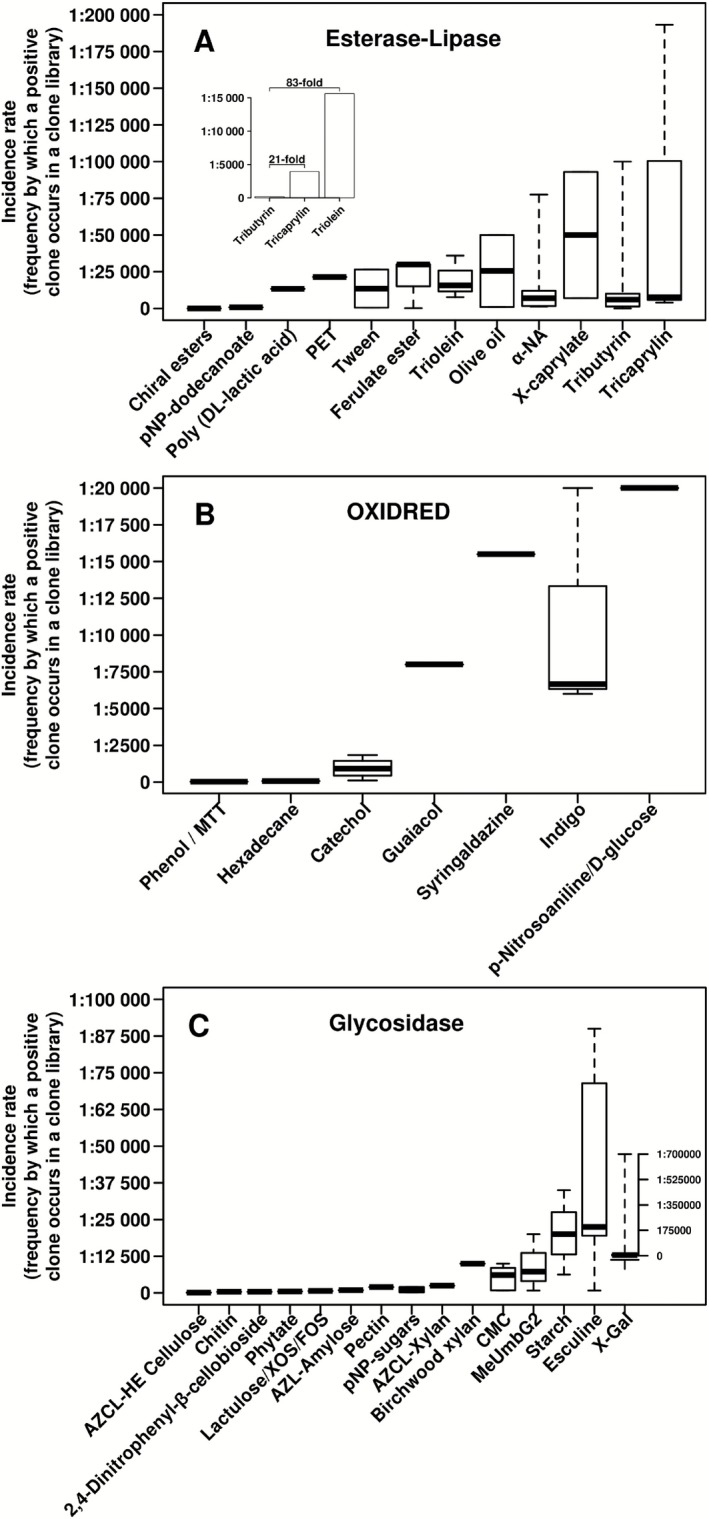
Box plots of the incidence rate of positive clones (referred to the total number of clones screened) with esterase‐lipase (A), oxidoreductase (B) or glycosidase (C) activity by substrate after naïve screens. The results are based on values published in previous metagenomic studies (see Fig. 1 legend), accounting for only those for which quantitative values are available. Note: As the incidence rate depends on the type of the clone library, only data regarding studies in which metagenomic fosmid clone libraries were screened were considered. Results of single references for (B) and (C) are given in Table S2. Abbreviations are as follows: AZCL, cross‐linked azurine; AZCL‐HE, azurine cross‐linked hydroxyethyl cellulose; CMC, carboxymethyl cellulose; X‐Gal, 5‐bromo‐4‐chloro‐3‐indolyl‐β‐D‐galactopyranoside; X‐caprylate, 5‐bromo‐4‐chloro‐3‐indolylcaprylate; α‐NA, α‐naphthyl acetate; OXIDRED, oxidoreductase; PET, polyethylene terephthalate; pNP‐dodecanoate, p‐nitrophenyl‐dodecanoate; pNP‐sugars, p‐nitrophenylsugars; XOS, xylo‐oligosaccharides.

Concerning the substrate‐dependent efficiency of screening programmes, a number of interesting patterns could be observed. Thus, it was demonstrated that the incidence rate decreased from 1:188 (2661 out of a total of 500 000 clones tested) to 1:3937 (127 clones) and 1:15 625 (32 clones) when the library was screened for esterase and lipase activity, respectively, using 1% (v/v) tributyrin, tricaprylin and triolein as the indicator substrates (Glogauer *et al*., 2011) (Fig. 4A, inset). This result implies that the enzymes with the lipase phenotype (most active against longer insoluble triglycerides such as triolein) were 83‐fold less abundant in this experiment than were those with an esterase (most active towards shorter triglycerides such as tributyrin) character. Additionally, among the common substrates used for the esterase/lipase screen, the methods using pH indicators resulted in a higher incidence rate (1:29) (Martínez‐Martínez *et al*., 2014), followed, to a lesser extent, by methods based on the utilization of indoxyl acetate (1:700) (Alcaide *et al*., 2013), nitrocefin [3‐(2, 4 dinitrostyrl)‐(6R,7R‐7‐(2‐thienylacetamido)‐ceph‐3‐em‐4‐carboxylic acid, E‐isomer)] (1:10 000) (Rashamuse *et al*., 2009), poly(DL‐lactic acid) (1:13 334) (Akutsu‐Shigeno *et al*., 2003; Okamura *et al*., 2010), tributyrin (1:15 478), α‐naphthyl acetate (1:19 925), polyethylene terephthalate (1:21 400) (Sulaiman *et al*., 2012), triolein/olive oil and rhodamine B (1:22 061) (Glogauer *et al*., 2011), Tween‐20 and CaCl_2_ (1:26 496) (Heravi *et al*., 2008; Okamura *et al*., 2010), methyl and ethyl ferulate (1:26 496) (Vieites *et al*., 2010), 5‐bromo‐4‐chloro‐3‐indolylcaprylate (1:50 000) (Li *et al*., 2008), and tricaprylin (1:68 279) (Tirawongsaroj *et al*., 2008), in that order (Fig. 4A). The aforementioned substrates represent some of the most commonly used substrates for which ample frequency data are available (from references given in Table S1). Note that actually at least 200 distinct substrate molecules have been successfully applied in assays for esterases/lipases biocatalysts at high throughput scale for selection in metagenomic clone libraries.

For oxidoreductases, among the seven distinct substrates that are commonly tested, phenol has been shown to achieve the highest relative number of positives (1:32), whereas p‐nitrosoaniline complemented with D‐glucose and used in combination for screening of α‐glucose dehydrogenase activity has been shown to exhibit the lowest hit rate (1:20 000) (Fig. 4B; see details in references given in Table S1). At least 15 distinct chromogenic and fluorimetric substrates, for which extensive frequency data are available, have been commonly and successfully employed for the screening of clones with glycosidase activity (from references given in Table S1). Cross‐linked azurine hydroxyethyl cellulose, a unique substrate for the measurement of endo‐cellulase, provided a major incidence rate (1:108) (93 out of a total of 10 000 clones tested) (Nguyen *et al*., 2012). In contrast, 5‐bromo‐4‐chloro‐3‐indolyl‐β‐D‐galactopyranoside (X‐gal), a common substrate for the screening of β‐galactosidase activity at high frequency, is the substrate providing in some cases the lowest number of positive hits (Wang *et al*., 2014) (1:700 000) (Fig. 4C).

Taken together, these findings suggest that in naïve screening programmes, the substrate may cause biases in the selection of the activities of interest. Clearly, the selection of the appropriate substrate is highly recommended. Recently, it has been demonstrated that the initial selection of active clones with general substrates followed by a more specific one is the most desired approach. This protocol has been successfully applied to the selection of (S)‐ketoprofen‐specific hydrolytic activities (Yoon *et al*., 2007). Here, the common esterase/lipase substrate α‐naphthyl acetate was employed as the initial screening substrate, followed by specific activity tests with (S)‐ketoprofen. Additionally, 5‐bromo‐4‐chloro‐3‐indolylcaprylate, whose hydrolysis produces blue colonies, was successfully applied as a primary substrate to screen 93 000 clones from the topsoil samples from vegetable soil. The positive clones (six in total) were further screened with a secondary substrate, pyrethroid, to identify one pyrethroid hydrolysing esterase, whose activity is difficult to test in the whole clone libraries (Li *et al*., 2008).

It is also plausible that screen conditions also produce biases in the rate of success, especially when the clone libraries were generated from microbial communities inhabiting extreme habitats. As an example, the incidence rate of positive clones for esterase/lipase activity for libraries originated from low‐salt habitats (1.1–38.6 g/kg total salinity) such as Lake Arreo (1:1152) or deep‐sea Matapan–Vavilov basin (1:667) (Martínez‐Martínez *et al*., 2013; Alcaide *et al*., 2015) was much higher than that in the same type of libraries from hypersaline environments, e.g. 1:2624 (for Medee Basin) and 1:5280 (Kryos Basin) (Alcaide *et al*., 2015). Since naïve screens are typically performed at 0.15 M NaCl, i.e. at salinities far below than in extreme hypersaline environments (e.g. 348 g/kg for Medee Basin), under these conditions extremozymes may exhibit lower activities, which leads to the reduction in hit rates. This has been recently demonstrated by examination of novel chitobiosidase from soil and by showing a better functioning at raised NaCl levels (Cretoiu *et al*., 2015). Therefore, selecting appropriate physical‐chemical parameters for naïve screens should carefully be considered in extensive screening programmes.

## Quantifying the success of sequence data mining for enzyme discovery

The recent revolution in high‐throughput DNA sequencing technologies has resulted in a significant reduction in the sequencing costs, leading to an explosion of the *in silico* data production and a dramatic expansion of the databases (Mende *et al*., 2012). In contrast, the pipelines for functional protein analysis operate at much lower rates and throughputs (Chistoserdova, 2014), opening the gap between the numbers of proteins/enzymes predicted *in silico* and those experimentally characterized in the lab with the proportion of the latter asymptotically approaching 0% (Anton *et al*., 2013; Bastard *et al*., 2014). There is a growing appreciation that this emerging gap between the high‐throughput metagenomic sequencing data and the experimentally characterized proteins must be considered (Bastard *et al*., 2014). For example, there are a few existing US National Institutes of Health (NIH)‐ and Department of Energy (DOE)‐sponsored initiatives to address this issue, including the large NIH‐funded Structural Genomics Consortium (supported since 2000) and the more recent COMBREX initiative (Anton *et al*., 2013), which looks into the systematic characterization of proteins from few dozens of reference microorganisms. These reference microorganisms include the best‐studied microbes *E. coli* and *Helicobacter pylori*, which – combined – have only 0.33% of their proteins characterized. Extending the knowledge to key industrial producer organisms beyond *E. coli* and *H. pylori*, such bacterial species of the genera Bacillus, Pseudomonas, Rhodobacter, Burkholderia, Streptomyces, eukaryotic models such as Saccharomyces and Pichia, fungi models such as Trichoderma, and model organisms in the domain Archaea, including methanogens, halophiles, Thermococcales and Sulfolobales, together with microorganisms residing in environmental samples, may be of interest. Through this investigation, one can produce data directly applicable to biotechnology while having important implications for our understanding of ecosystem and protein functioning.

Next‐generation sequencing for the identification of enzymes in metagenomes is therefore becoming increasingly important to generate enzyme collections (Wang *et al*., 2010; Nyyssönen *et al*., 2013) because it provides a rapid and cost‐efficient technology for enzyme discovery. Thus, a number of bioinformatics tools have been designed for the rapid pre‐selection of enzyme candidates after examining the sequence data obtained from different platforms. Predicted protein‐coding genes are filtered according to their similarity with general protein databanks (UniProt, NCBI NR), or to their similarity to conserved domains according to the Pfam and Common Domains database (e.g. Fajardo and Fiser, 2013), or specific updated enzyme sequence resources, such as the Carbohydrate‐Active Enzyme (CAZyme) (Cantarel *et al*., 2009), the Lipase‐Esterase (Barth *et al*., 2004), the Laccase (Sirim *et al*., 2011), the PeroxiBase (Fawal *et al*., 2013), the metallo‐β‐lactamase (Widmann and Pleiss, 2014), the amine transaminases (Steffen‐Munsberg *et al*., 2015) and the AromaDeg (Duarte *et al*., 2014) databases.

In a second step, it is possible to obtain the general features of the proteins (mass, pKa, motifs, existence or absence of a secretion signal) for each type of sequences, and the protein sequences can be analysed in detail to identify the domains or motifs that are specific for the desired activity or structurally classified by the active site modelling and clustering method (Marsh *et al*., 2012). Further, selected genes that encode enzymes of interest may be subjected to high‐throughput expression analysis for their subsequent production and characterization; this approach, the so‐called synthetic metagenomics, is being extensively used (Wang *et al*., 2010; Dougherty *et al*., 2012; Gladden *et al*., 2014).

Bioinformatic tools applied to the screening of sequence data have been successfully used to identify epoxide hydrolases (Jiménez *et al*., 2015), haloalkane dehalogenases (Barth *et al*., 2004) and carbohydrate esterases (Tasse *et al*., 2010). Recently, Schallmey and colleagues (2014) used specific sequence motifs to identify 37 novel halohydrin dehalogenases, very rare promiscuous enzymes, in public databases. All of the enzymes were expressed, and their catalytic performances were successfully tested. However, one of the problems in using such an approach, other than the inconvenience of identifying entirely new enzymes with sequences far distant from those in repository databases, the quality of the assembly and the problems in protein expression, is the limited rate of success. As an example, Schallmey and colleagues (2014) retrieved only 37 novel enzymes that catalysed halohydrin dehalogenase reactions from 35 448 available public sequences. This means that they had an incidence rate of 1:958, which is similar to the rate that is commonly achieved by naïve screens. For comparison, the screening of 704 000 clones from microbial communities isolated from human faecal material identified 310 positives. This was followed by the pyrosequencing of the insert, and a total of 662 complete genes were predicted. Of these, 73 were CAZyme proteins, making an incidence rate of 1:9 (1 gene encoding an enzyme of interest per 9 total genes). This number is much more favourable than that obtained from selection via direct DNA sequencing or the use of public databases.

## Occurrence of industrial enzymes across genomes

One further question that may arise is how the incidence rate during naïve or *in silico* screen programmes in metagenome libraries or meta‐sequences related to incidences of gene targets within bacterial, archaeal or even fungal genomes. Is there any bias in the screen efficiency due to the differences in the occurrence of particular genes in microbial genomes? To answer this question, we revised the bibliographic records for the genes encoding the six most popular industrially relevant enzymes mentioned above: acylases, phosphatases, proteases, oxidoreductases, glycosyl hydrolases and lipases/esterases. Comparative genomics has revealed that glycosyl hydrolase‐related genes comprise 0.05–6% (referred to the total number of genes) in bacterial genomes (Coutinho *et al*., 2003), and up to *c.* 1.7% in archaeal (Werner *et al*., 2014) and 1.5% in fungal (Islam *et al*., 2012) genomes. This indicates high differences in gene abundance across genomes. Similar scenario can be seen with esterases/lipases, ubiquitous enzymes widespread in nature whose frequency have been shown to range from at least 0.05% to 0.35% in bacterial and fungal genomes (Wang *et al*., 2010; Barriuso *et al*., 2013). For proteases, bacterial and archaeal genomes contain 4–29 per genome (Tripathi and Sowdhamini, 2008), while in fungal genomes 1–178 per genome (Budak *et al*., 2014). For phosphatases, the number ranges from 0.06% to 7.5% referred to the total genes (Galperin *et al*., 2010). No data are available for acylases and oxidoreductases.

Taken together, it is plausible that biases in the screen efficiency may be also partially due to the fact that the enzyme class of interest occurs sparsely in the genomes of microbial members residing in an environmental sample. Clearly, the community structure and metagenome sequence diversity and divergence may thus play a role in screen programmes.

## Success stories for introducing environmental enzymes into the market

Funding agencies, worldwide companies and laboratories have adopted a number of actions, and research activities are ongoing to decrease the time frame for enzyme identification (see comments below) and the very expensive and time‐consuming biocatalysts optimization phase while increasing the efficiency of the processes. However, there are very few cases in which a new environmental biocatalyst has been translated to a process in recent times (Fernández‐Arrojo *et al*., 2010). In fact, only few metagenomics‐based enzyme products have been patented and translated to market. Having said that, industrial enzymes will have to be novel and not found in the patented literature, since this is the only chance for new enzymes to make an impact ‘beyond the state of the art’. In this sense several metagenomic enzymes have been patented, e.g. nitrile hydratases (EP2369009A3), soil metagenome‐derived gene wes (WO2013125808A1), caw rumen‐derived esterases (EP04015920.4), cellulases (EP04015680.4) and laccases (GB01P006EP), and an esterase from uncultured microorganisms able to degrade terephtalate esters, important component of bioplastics (WO 2007017181). It is important to note that independently of the novelty of the sequence encoding an enzyme, the key is the application. The use of the new enzyme for exactly the same application would violate the ‘inventive’ portion of any new IP to be generated, while violation of the ‘novelty’ is permitted.

## Final considerations: backbones of interest for finding marketable enzymes

**Figure 5:**
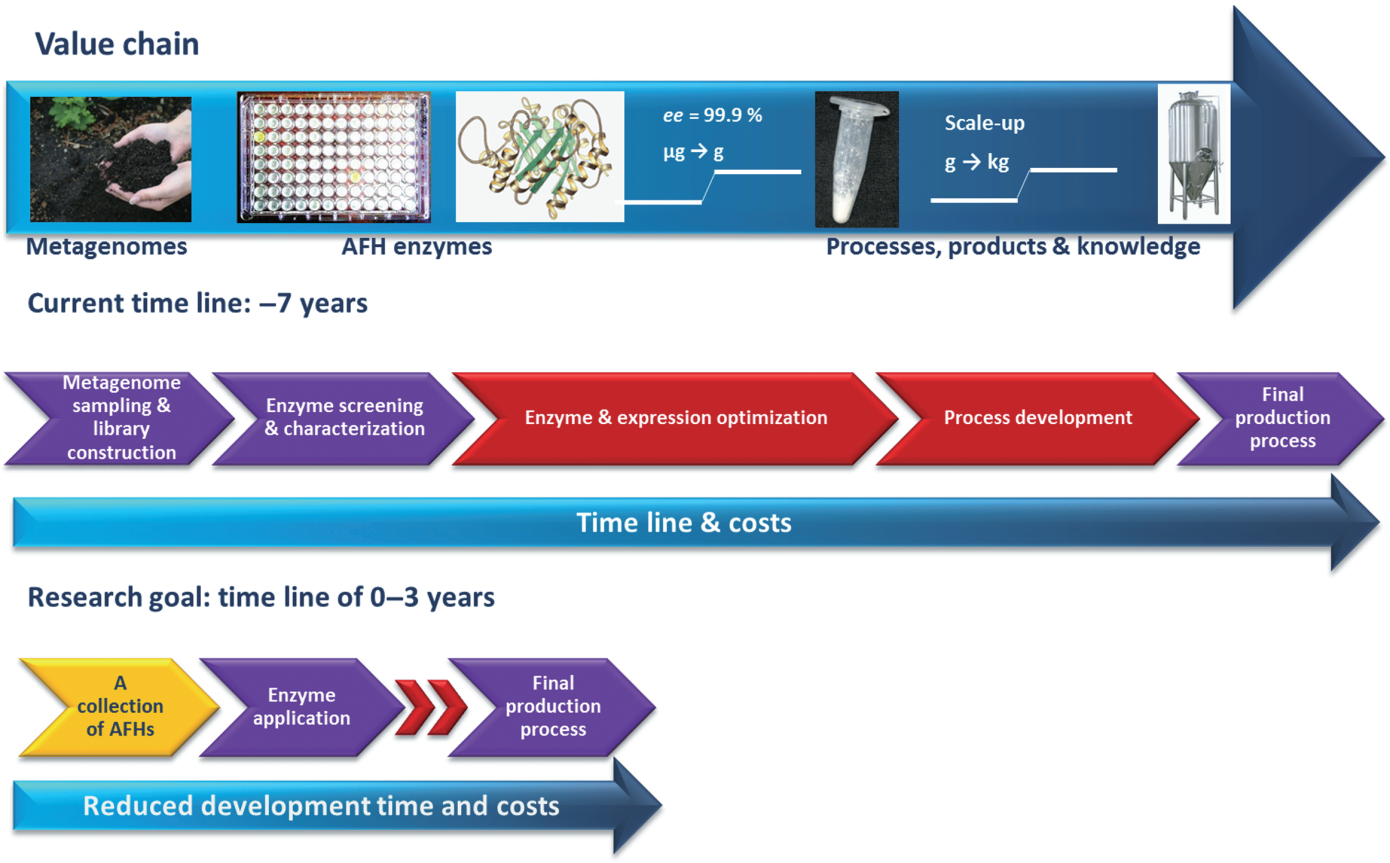
The value chain from enzyme identification to biocatalytic process implementation is shown. It now takes at least 5–7 years to develop a new enzyme‐based production process. The most time‐ and cost‐consuming steps are the multifactorial optimization of the biochemical enzyme properties and the expression optimization to achieve high biocatalyst yields. New process development must be completed for each new biocatalyst. Intensive and optimized metagenomic screening programmes will shorten this process (expected to be up 3 years) by providing an enzyme collection of AFHs (‘all‐round frequent hitters’) with promiscuous activities that can be directly applied to existing or new processes.

It usually takes several (typically, approximately seven) years from the time when a gene is identified until the industrial process is established (Fig. 5) (Fernández‐Arrojo *et al*., 2010). This is not only because of the technical issues around the process of enzyme discovery, but also because enzymes only end up in industrial processes if they comply with the industrial criteria. They included the following: (i) harsh and broad reaction conditions such as a high substrate load (necessary to reduce the costs to be competitive), broad range of temperatures (at least should be stable at room temperature for a period of time as also storage might be an additional issue – think in detergent enzymes applied in warmer countries), broad range of pHs, water‐deficient reaction conditions, very high solvent concentrations (which for example might be necessary for subsequent downstream processing) and process stability (e.g. active for 12–24 h) (Spickermann *et al*., 2014; Zuhse *et al*., 2015); and (ii) the high stereoselectivity and high turnover rates (Singh, 2010). As example, enzymes applied in feed must be thermostable (due to the pelleting process) and must be stable or active at low pH (stomach of animals) (Viader‐Salvadó *et al*., 2010). Also, additives such as salts to a high concentration can be used as additives for enzyme stabilization under industrially relevant conditions, and therefore the halophilic enzymes, such as alcohol dehydrogenases, may be desired for certain applications (Spickermann *et al*., 2014). Clearly, novel backbones from metagenomes might meet these requirements. One further aspect should be considered: an enzyme will enter to the market if discovered in a reasonable time frame; actually, 3 years is the desired time frame for the introduction of new enzymes into the market (Fig. 5).

To improve the selection process of industrially relevant enzyme, a number of protocols have been suggested. The first one is based on the fact that a correlation between gene expression and the turnover rate for substrate transformation has been observed (Helbling *et al*., 2012). Accordingly, enrichment procedures with model (proxy) substrates relevant to industry under the desired conditions might be useful in designing more efficient industrially relevant enzyme discovery approaches (Jacquiod *et al*., 2013; Verastegui *et al*., 2014; Vester *et al*., 2014). Clearly, the examination of cDNA or metaproteomes by shotgun metatranscriptomic and proteomic approaches, rather than direct DNA sequencing, could be used to query the most active clones or enzymes. The identification depends heavily on gene and protein abundance, and although we are aware that a large part of the transcriptome and proteome remains unseen, it can be assumed that the identified genes and enzymes might represent the predominant (in terms of dosage per cell and expression levels) and the most active genes and enzymes under the tested conditions. A further evaluation of enzyme performance under multiple conditions using high‐throughput parameter (Kunze *et al*., 2014) may allow sorting out the possibility to identify highly active, efficient and promiscuous (Pandya *et al*., 2014) enzymes under real or close‐to‐real process conditions, independently of the further optimization phase to which the enzyme can be subjected (Bornscheuer *et al*., 2012).

## Conflict of Interest

The authors declare that they have no competing interests.

## Supporting information


**Table S1.** List of sites worldwide where metagenomic studies have been performed. These sites corresponds to those summarized in Fig. 1. The exact GPS (latitude and longitude) location of sites together with appropriated references and site characteristics are specifically described. Whether the habitats have been subjected to direct sequencing [for community structure analysis and gene content by high throughput (HTP) sequencing] or enzyme screening (analysis of target genes either by naïve or *in silico* screens) is also cited.
**Table S2.** Results of single references for the incidence rates of positive clones or enzymes. Examples are provided for the screening of oxidoreductase and glycosidase activity by using multiple substrates after naïve screens. The number of clones tested, the number of positive clones, the incidence rate, the substrate used and the reference are given. For abbreviations, see Fig. 4 legend.
